# Ketoglutaric acid can reprogram the immunophenotype of triple-negative breast cancer after radiotherapy and improve the therapeutic effect of anti-PD-L1

**DOI:** 10.1186/s12967-023-04312-2

**Published:** 2023-07-12

**Authors:** Hongpei Tan, Jiahao Liu, Jing Huang, Yanan Li, Qiongxuan Xie, Yuqian Dong, Ze Mi, Xiaoqian Ma, Pengfei Rong

**Affiliations:** 1 Department of Radiology, Third Xiangya Hospital, Central South University, No. 138 Tongzipo Road, Changsha, 410013 Hunan China; 2 Department of Anesthesiology, Zhuzhou Central Hospital, Zhuzhou, 412000 China; 3 Department of Oncology, Xiangya Hospital, Central South University, Changsha, 410000 China

**Keywords:** Ketoglutaric acid, TNBC, PD-L1, Radiotherapy, Immunogenic death, Autophagy

## Abstract

**Background:**

Great progress has been made in applying immunotherapy to the clinical treatment of tumors. However, many patients with triple-negative breast cancer (TNBC) cannot benefit from immunotherapy due to the immune desert type of TNBC, which is unresponsive to immunotherapy. DMKG, a cell-permeable derivative of α-KG, has shown potential to address this issue.

**Method:**

We investigated the effects of combining DMKG with radioimmunotherapy on TNBC. We assessed the ability of DMKG to promote tumor cell apoptosis and immunogenic death induced by radiotherapy (RT), as well as its impact on autophagy reduction, antigen and inflammatory factor release, DC cell activation, and infiltration of immune cells in the tumor area.

**Result:**

Our findings indicated that DMKG significantly promoted tumor cell apoptosis and immunogenic death induced by RT. DMKG also significantly reduced autophagy in tumor cells, resulting in increased release of antigens and inflammatory factors, thereby activating DC cells. Furthermore, DMKG promoted infiltration of CD8 + T cells in the tumor area and reduced the composition of T-regulatory cells after RT, reshaping the tumor immune microenvironment. Both DMKG and RT increased the expression of PD-L1 at immune checkpoints. When combined with anti-PD-L1 drugs (α-PD-L1), they significantly inhibited tumor growth without causing obvious side effects during treatment.

**Conclusion:**

Our study underscores the potential of pairing DMKG with radioimmunotherapy as an effective strategy for treating TNBC by promoting apoptosis, immunogenic death, and remodeling the tumor immune microenvironment. This combination therapy could offer a promising therapeutic avenue for TNBC patients unresponsive to conventional immunotherapy.

**Graphical Abstract:**

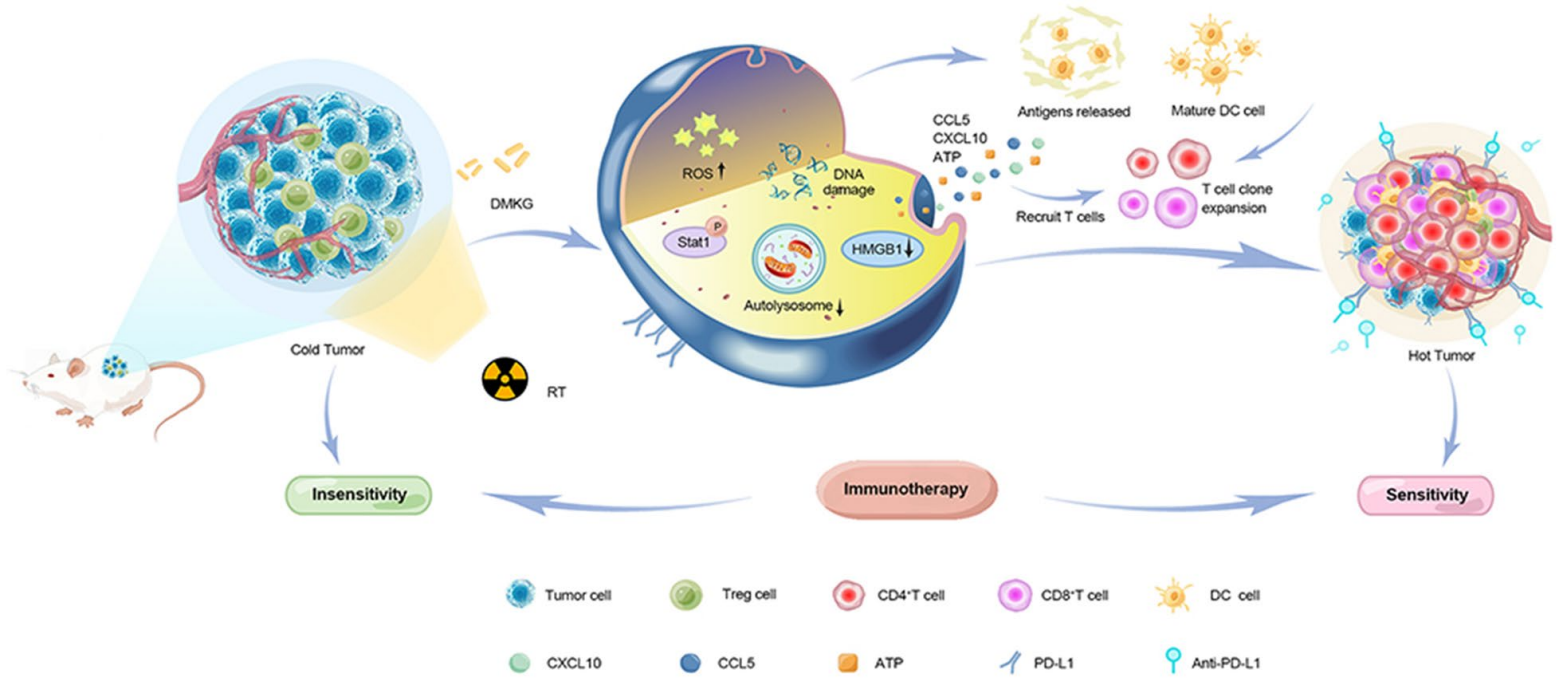

**Supplementary Information:**

The online version contains supplementary material available at 10.1186/s12967-023-04312-2.

## Introduction

Globally, cancer is a major public health problem, and in the United States it ranks second in terms of cause of death. In the United States, breast cancer accounts for the largest proportion of new cancer cases [1]. Triple-negative breast cancer (TNBC) emerges as the subtype with the most adverse prognosis compared to other breast cancer types [2, 3]. Characterized by its aggressive invasiveness and a heightened propensity for distant metastasis, the median survival span post-metastasis is alarmingly limited to approximately 13.3 months [4]. In the treatment of breast cancer, radiotherapy is often used for localized control, but the survival prospects for patients are still not ideal [5, 6]. While recent advancements in immunotherapy offer promising outcomes in TNBC management, treatment resistance remains a notable obstacle [7]. This resistance might be attributable to TNBC's immune desert phenotype, which poses a significant barrier to immune cell infiltration into the tumor microenvironment [8, 9]. Some studies have used radiation to create a proinflammatory environment in the tumor area to increase the efficacy of immunotherapy, but the therapeutic effect on TNBC is still not ideal [10, 11]. A possible important reason is that although radiotherapy can lead to infiltration of inflammatory cells, additionally, it can lead to an increase in local myeloid-derived suppressor cells and regulatory T cells; Furthermore, the autophagy surge triggered by radiotherapy can impair DC cell activation, thereby influencing the impact of immunotherapy [12–15]. Consequently, strategic modulation of the post-radiation tumor immune microenvironment to augment the immunotherapeutic response carries considerable implications for clinical interventions in TNBC.

Numerous studies have probed into the reformation of the tumor microenvironment and the enhancement of therapeutic sensitization [16, 17]. However, the clinical application of most drugs employed in these studies has been curtailed predominantly due to biosafety concerns, largely limiting them to the domain of preclinical investigations. Consequently, employing commonplace metabolic products to transform the tumor microenvironment and potentiate therapeutic outcomes emerges as a prospective strategy. As a key component of the tricarboxylic acid cycle, alpha-ketoglutarate (α-KG) is a molecule that plays an important role in a number of physiological processes, including lipid production, reducing oxidative stress, modifying proteins, and inducing cell death. Current evidence positions α-KG as a promising candidate for anti-cancer strategies due to its perceived therapeutic potential [18, 19]. Particularly in the context of P53-deficient malignancies, an accumulation of α-KG might impede malignant progression by stimulating differentiation of tumor cells [20]. Additionally, an escalated α-KG/succinate ratio, achieved by disrupting mitochondrial complex I, has demonstrated efficacy in suppressing tumor growth [21]. Empirical findings suggest that exogenous AKG supplementation confers health benefits, thereby establishing its sound biosafety profile and underscoring the merit of its comprehensive exploration in oncological applications [22]. In the present study, we have scrutinized the effect of combining AKG with radioimmunotherapy on the tumor and immune microenvironment, intending to provide a theoretical underpinning for the preclinical utilization of AKG in tumor interventions.

## Methods

### Cells

Breast tumor cells (4T1, MBA-MD-231), Human umbilical vein endothelial cells (HUEVCs) and mouse bone marrow derived dendritic cells (DC2.4) was presented by Central South University's School of Pharmacy (Changsha, China). HUVECs were cultured and grown in corresponding supplement kit. Other Cells were cultured in RPMI-1640 medium containing 10% fetal bovine serum, 1% penicillin/streptomycin solution, and kept at 37 °C under a 5% CO2 atmosphere.

### Animals

This study was approved by the Ethics Committee of the Third Xiangya Hospital, Central South University, in accordance with the Helsinki Declaration of 1975 (as revised in 2008). Female BALB/c mice (8–9 weeks old) were provided by the Department of Laboratory Animals at Central South University (Changsha, China) and were used to establish tumor models by subcutaneously injecting 2 × 10^6^ 4T1 cells into the right side of the back of each mouse.

### ROS detection

MBA-MD-231 cells were seeded in confocal dishes at a density of 5 × 10^4^ cells per dish and allowed to incubate overnight. Following this, the cells were treated with 10 mM DMKG and 4 Gy of X-ray radiation. After additional incubation for 18 h, DCFH-DA (10 μM) was added for 30 min of staining, and Hoechst 33,342 was used for 20 min to stain the nuclei. The cells were then imaged using confocal laser scanning microscopy and the intracellular ROS levels were quantified via flow cytometry. The irradiator used in this study is PXI X-RAD 225(USA).

### DNA damage detection

MBA-MD-231 cells were seeded in confocal culture dishes at a density of 5 × 104 cells/dish and incubated overnight. After treatment with DMKG (10 mM) and a 4 Gy dose of X-ray, the cells were further incubated for 18 h before being fixed with 4% paraformaldehyde and treated with 0.2% Triton X-100 for 15 min. The cells were then incubated with a γ-h2ax primary antibody and Alexa Fluor 555-labelled donkey anti-rabbit IgG, before being counterstained with DAPI (1 μg/ml) for 15 min. Finally, the morphology of the cells was observed using confocal laser microscopy.

### Cyto-ID autophagy detection kit assay

MDA-MB-231 cells were treated in the specified way. The cells were incubated for 30 min in DMEM (Enzo, ENZ-KIT175-0050) without phenol red, stained with Hoechst nucleus for 10 min, and then washed with PBS. A confocal microscope was used for image acquisition.

### Detection of immunogenic cell death (ICD)

A study was conducted to evaluate the induction of ICD by examining calreticulin (CRT) exposure, levels of the protein HMGB1, and the release of ATP. The 4T1 cells were seeded in 24-well plates and treated in the described manner. A standard enzyme-linked immunosorbent assay kit was used to measure HMGB1 and ATP levels in the culture supernatant.

### DC maturation study

24 well plates were seeded with DC2.4 cells and incubated overnight. 4T1 cells were treated with DMKG (10 mM, 6 h) and 4 Gy X-ray, cleaned with PBS, and then continued to culture for 12 h. The supernatant was then collected for the treatment of the DC2.4 cells. Next day, the DC2.4 cells were harvested, stained with the corresponding antibody, and then analysed by flow cytometry. CD11c was used to differentiate DC cells. CD40, CD80, CD86 and MHCII were used to evaluate DC cell activation.

### Enzyme-linked immunosorbent assay

For tumor tissues, the environment was maintained at 4 °C, and the tumor was washed in PBS. A homogenate of tumor tissue is obtained by mashing the tissue, in the next step, the supernatant was collected after centrifugation at 1500 g for 10 min. The cells in vitro were placed in a six-well culture dish. After the cells were treated following the corresponding methods, the cell supernatant was collected and centrifuged at 1500 g for 15 min for detection.

### In vivo antitumor therapy

We randomly divided the tumor-bearing mice into eight groups (n = 5): (1) PBS group; (2) RT group; (3) DMKG group; (4) RT + DMKG group; (5) anti PD—L1 group; (6) RT + anti PD—L1 group; (7) DMKG + anti PD—L1 group; and (8) RT + DMKG + anti PD—L1 group. Start treatment when the initial tumor volume of the mouse is about 100mm^3^. A dose of 4 Gy X-ray radiation was administered to the mice on days 0 and 4, DMKG (500 mg/kg) was injected locally into the tumor on Days 0 and 4, and αPD-L1 (100ug) was injected intraperitoneally on Days 3 and 5. A measurement of the tumor size and weight was taken every two days. The in vivo experiment was carried out twice, one was to take mouse tumors on the tenth day for further digestion for flow cytometry analysis, and one was to take the mouse tumors for pathological staining on the 16th day, and blood was collected to assess changes in liver and kidney function.

### Tumor-infiltrating immune cell analysis

As mentioned above, mice were divided into groups with five mice in each group. The tumors of mice were taken on the seventh day of treatment to investigate the changes of immune microenvironment. Single cells were isolated by slicing the tumors into small pieces and digesting them at 37 °C for 1 h. The cells were stained with the corresponding antibody. Flow cytometry (FACSVerse, BD, USA) was then used to analyze the samples. FOXP3 and CD4 were used to evaluate the Treg cell subsets. IFN-γ and granzyme B were used to evaluate CD8 + T cell activation. All reagents, antibodies and RNA primers used in the study are provided in the Supplementary Materials.

### Statistical analysis

The mean + standard deviation (SD) is used to report all quantitative results. The differences among multiple groups were assessed using one-way analysis of variance (ANOVA).

## Results

### DMKG has a synergistic antitumor effect with radiotherapy

**Fig. 1:**
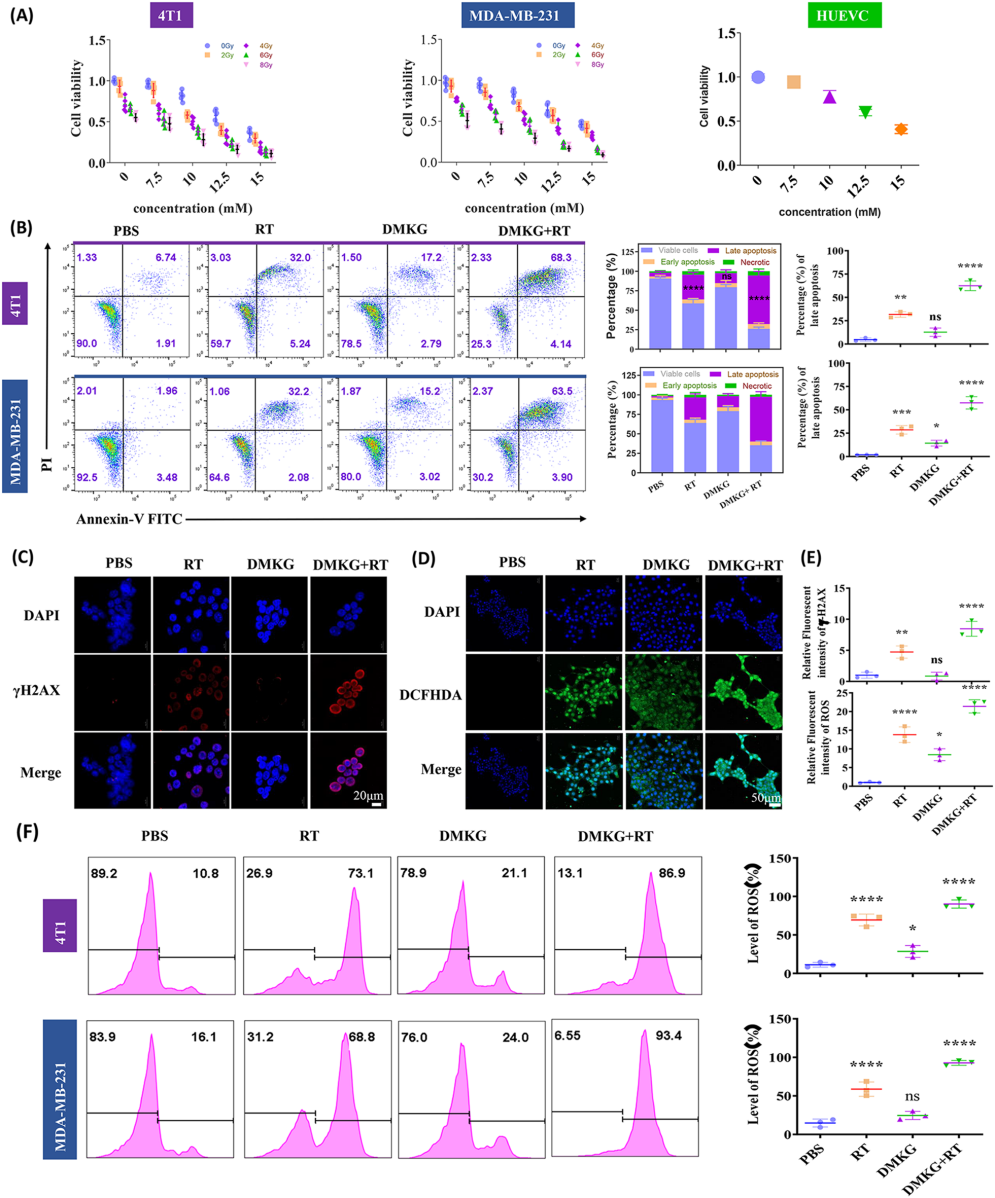
**A** Changes in the viability of 4T1, MDA-MB-231 and HUEVC cells under different concentrations of DMKG and different radiation doses. **B** Apoptosis of 4T1 and MDA-MB-231 cells under different interventions, detected by flow cytometry. **C** DNA damage of 4T1 cells indicated by immunofluorescence staining of γ-H2AX after various treatments. **D** Intracellular ROS detection in 4T1 cells by DCFH-DA after various treatments. **E** Fluorescence quantization results of ROS and γH2AX. **F** Flow cytometry detection of intracellular ROS levels and corresponding quantitative plots. The concentration of DMKG was 10 mM. The radiation dose was 4 Gy. Scale bar was shown in the figure

Dimethyl-α-ketoglutarate (DM-αKG, a cell-permeable analogue of α-KG) was used to treat MDA-MB-231 and 4T1 cells. Different concentrations of DMKG and different radiation doses were used to treat cancer cell lines. Tumor cell viability was measured by CCK8 at 48 h (Fig. 1A), and it was found that DMKG significantly enhanced the efficacy of radiotherapy and inhibited the viability of tumor cells. Based on the effect of DMKG in normal cell lines (Fig. 1A) and our previous studies [23], we selected a DMKG concentration of 10 mM and a radiotherapy dose of 4 Gy for further study. Colony formation experiments showed that DMKG and radiotherapy had inhibitory effects on breast cancer cells (Additional file 1: Figure S1). Flow cytometry was used to detect the apoptotic effect of DMKG and radiotherapy on breast cancer cell lines. Both can promote apoptosis, and in combination they can better promote the apoptosis of breast cancer cells (Fig. 1B). Since radiotherapy mainly damages the nucleus through reactive oxygen species and thus inhibits tumors, we showed by immunofluorescence the degree of nuclear damage (Fig. 1C) and the level of intracellular reactive oxygen species (Fig. 1D) under the intervention of radiotherapy and DMKG. The results showed that radiotherapy significantly increased intracellular reactive oxygen species and the degree of nuclear damage, while DMKG mainly promoted the production of reactive oxygen species, and the combined use of both significantly increased nuclear damage and the level of intracellular reactive oxygen species (Fig. 1E). Flow cytometry further verified the intracellular reactive oxygen species levels (Fig. 1F).

### DMKG promotes the cellular immunogenic death caused by radiotherapy

**Fig. 2:**
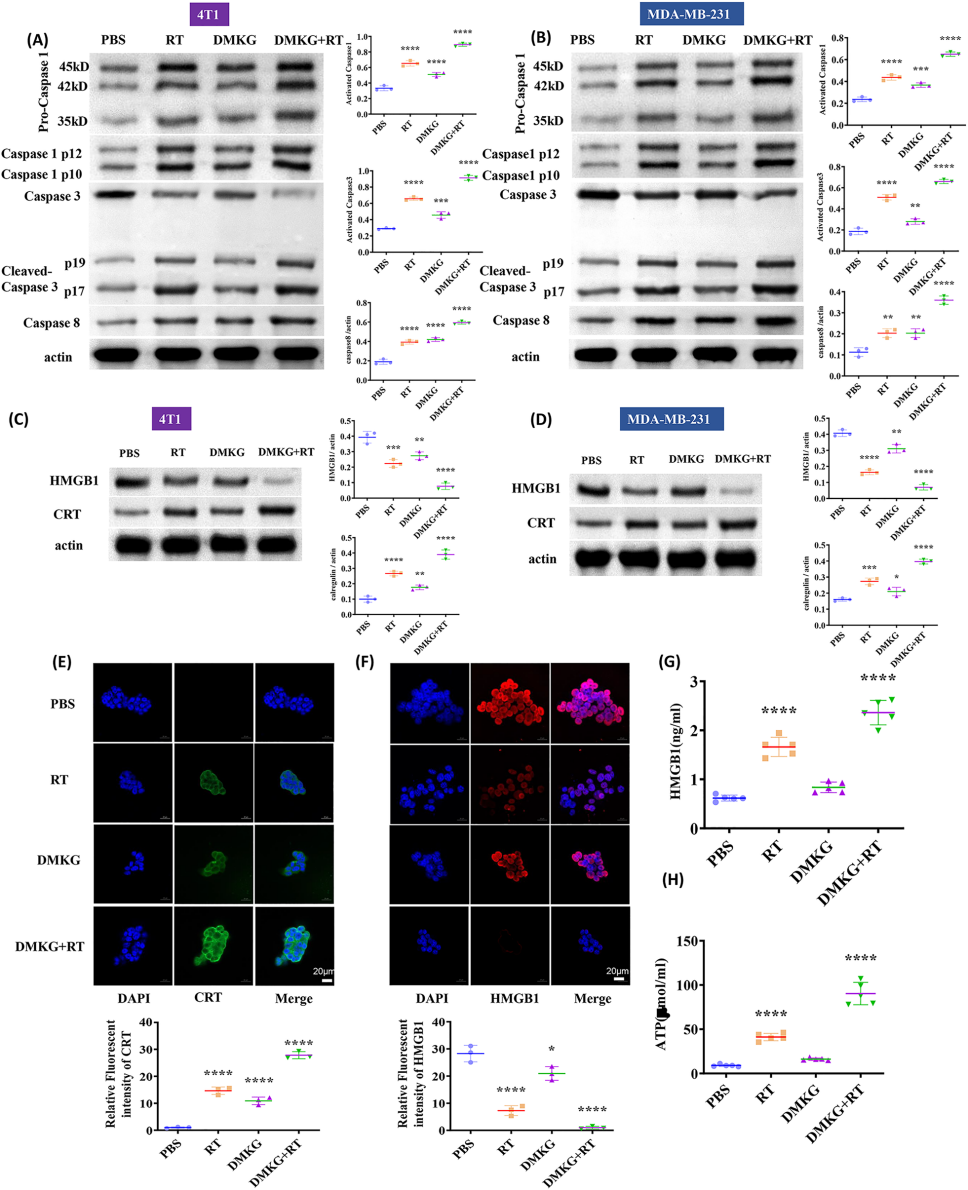
Expression and activation of caspase1, 3, and 8 in 4T1 (**A**) and MDA-MB-231 (**B**) cells under different treatments. Protein levels of HMGB1 and CRT in 4T1 (**C**) and MDA-MB-231 (**D**) cells under different treatments. Immunofluorescence staining of CRT (**E**) and HMGB1 (**F**) in 4T1 cells after different treatments. HMGB1 (**G**) release and ATP (**H**) secretion from 4T1 cells after different treatments (measured by an ELISA kit)

First, we detected the expression levels of caspase1, 3, and 8 by Western blotting. Radiotherapy activated caspase1, 3, and 8, while DMKG mainly activated caspase1 and caspase8 (Fig. 2A, B). Since related studies have shown that radiotherapy can promote immunogenic death of tumor cells, immunogenic death can be observed by detecting CRT and HMGB1. Our results showed that radiotherapy can promote the expression of CRT and the extracellular release of HMGB1, and that DMKG can significantly promote immunogenic death caused by radiotherapy (Fig. 2C, D). The fluorescence results further confirmed the results (Fig. 2E, F). Finally, by using ELISA we detected the release of ATP and the level of HMGB1, which further showed that DMKG can promote the immunogenic death caused by radiotherapy (Fig. 2G, F).

### DMKG inhibits autophagy induced by radiotherapy

**Fig. 3:**
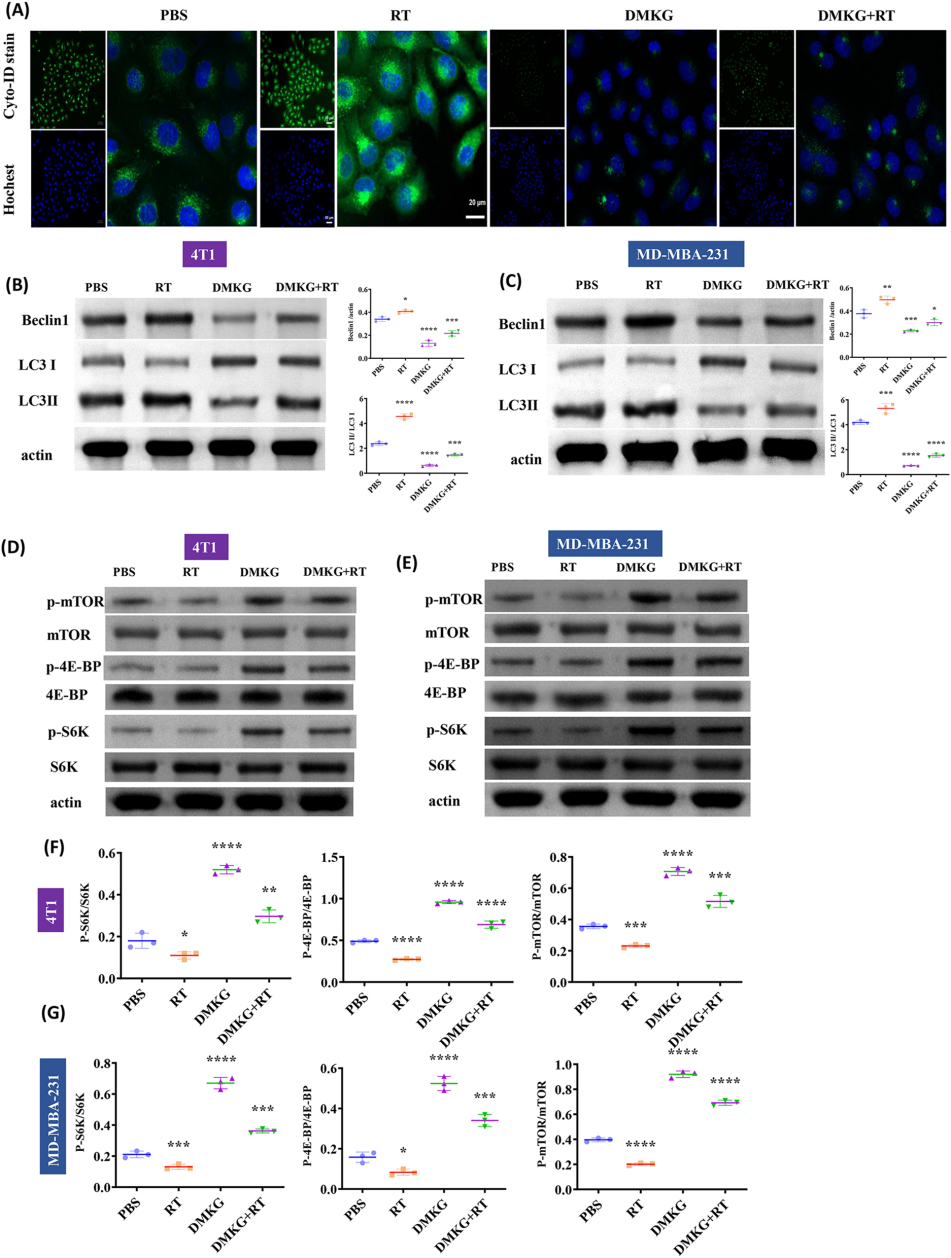
**A** The autophagy level of 4T1 cells under different treatment methods was detected and stained with a Cyto-ID autophagy detection kit 2.0 (Enzo). Expression levels of autophagy-related proteins in 4T1 (**B**) and MDA-MB-231 (**C**) cells under different treatments. The expression levels of several core components involved in the mTOR signaling pathway under different treatments [4T1(**D** and **F**), MDA-MB-231(**E** and **G**)]

Tumor can promote drug resistance through autophagy, which is also a primary way for tumor to resist radiotherapy [24]. We showed the formation of autophagosomes by fluorescence staining. The results showed that radiotherapy significantly increased the level of autophagy, while DMKG significantly inhibited tumor autophagy (Fig. 3A). Then, we detected the levels of LC3 and beclin1 by WB to further verify the effects of radiotherapy and DMKG on autophagy, the ratio of LC3I to LC3II indicates the change of autophagy. The results show that RT promotes LC3II, but DMKG reduces LC3II, indicating that RT can promote autophagy while DMKG inhibits autophagy. We also further verified by the expression of beclin1 (Fig. 3B, C). Since mTOR is an important pathway in regulating autophagy, inhibition of mTOR will promote autophagy, so we detected the activation and expression of related core proteins. The results show that RT can reduce the phosphorylation of mTOR-related proteins, while DMKG can promote the phosphorylation of mTOR-related proteins, which may be the possible mechanism of DMKG inhibiting autophagy induced by radiotherapy (Fig. 3D–G).

### DMKG promotes changes in the immune microenvironment

**Fig. 4:**
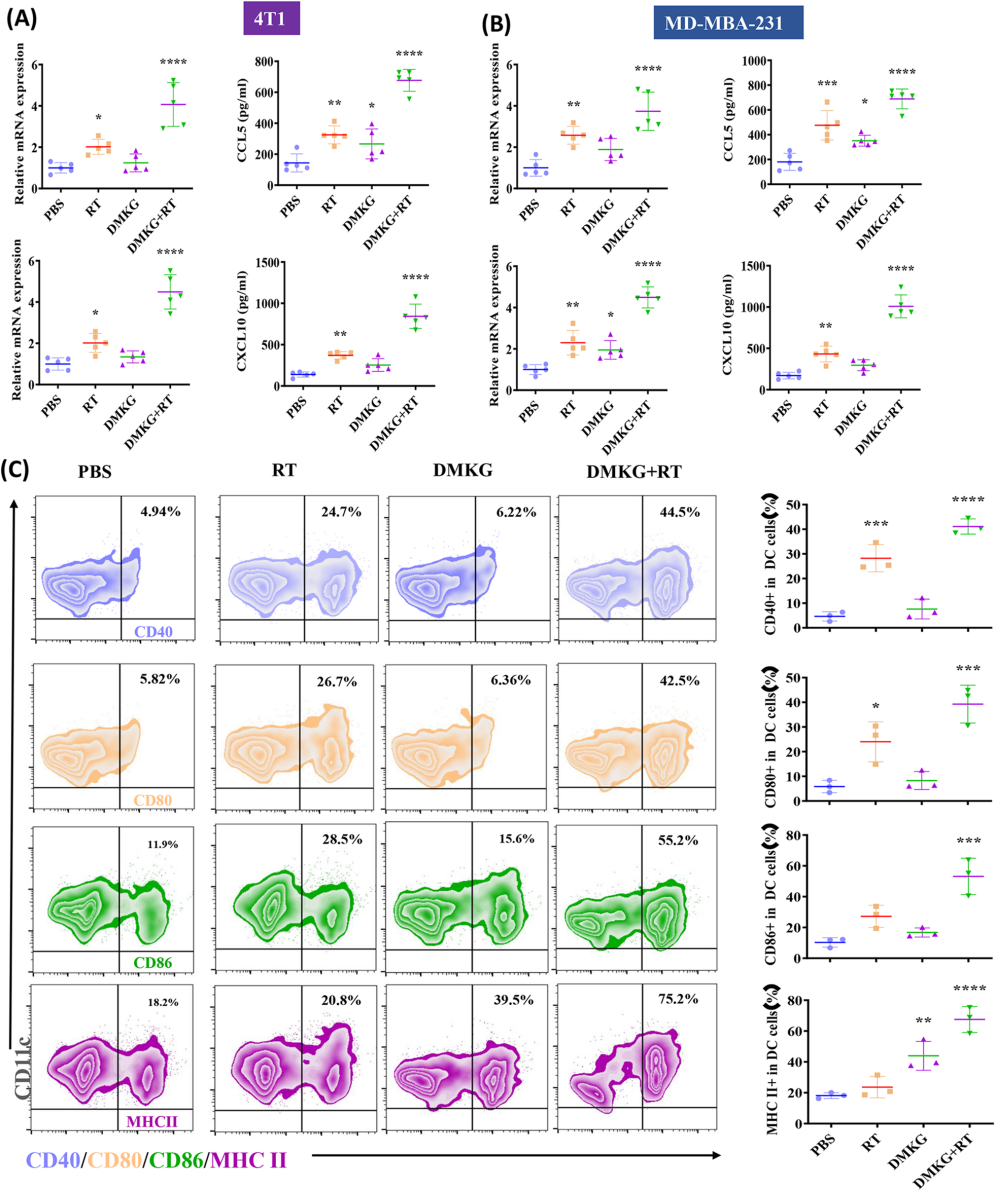
Expression of CCL5 and CXCL10 mRNA by RT‒qPCR and ELISA quantification of CCL5 and CXCL10 protein levels in the supernatants of 4T1 (**A**) and MDA-MB-231 (**B**) cells under different treatments. (**C**) Flow cytometry showed the activation of 4T1 cell culture medium on DCs under different treatments

To further study the effects of radiotherapy and DMKG on the immune microenvironment, we detected the RNA levels of the proinflammatory cytokines CCL5 and CXCL10 and their protein content in the supernatant. Radiotherapy combined with DMKG significantly promoted the release of inflammatory cytokines (Fig. 4A, B). The activation of DCs is very important for tumor immunoregulation. We treated DC cells with tumor culture supernatant, and then the activation of DC cells was detected by flow cytometry. The results showed that treating tumors with radiotherapy and DMKG can significantly promote the activation of DC (Fig. 4C), the activation of DC cells was further verified in vivo (Additional file 4: Figure S4).

### Radiotherapy and DMKG affect the expression of immune checkpoints

**Fig. 5:**
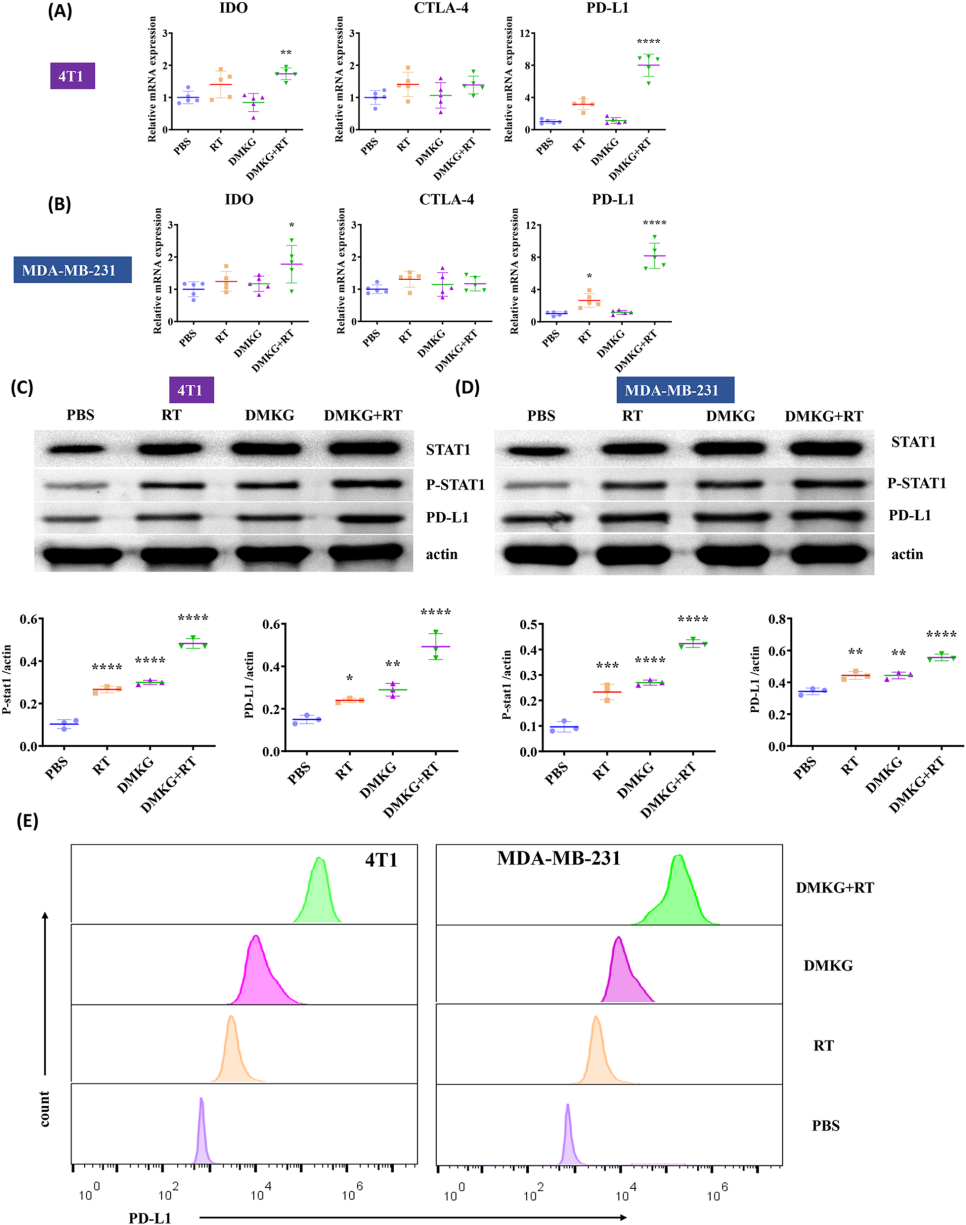
RNA expression levels of IDO, CTLA-4 and PD-L1 in 4T1 (**A**) and MDA-MB-231 **B** cells under different treatments. STAT1 and STAT1 phosphorylation and PD-L1 protein expression levels in 4T1 (**C**) and MDA-MB-231 **D** cells under different treatments. **E** Flow cytometry of PD-L1 expression in breast cancer cells under different treatments

The function of T cells is affected by immune checkpoint expression. We detected the RNA expression levels of IDO, CTLA-4 and PD-L1. The results show that radiotherapy and DMKG increased the expression of PD-L1 (Fig. 5A, B). Since related studies have shown that STAT1 can affect the expression of PD-L1 [25, 26], In addition, relevant literature has also confirmed that radiotherapy can promote the expression of PD-L1 through STAT1 [27]. We confirmed by WB that radiotherapy and DMKG can activate STAT1 and enhance the expression of PD-L1 (Fig. 5C, D). The expression of PD-L1 was further verified by flow cytometry (Fig. 5E). Subsequently, fluorescence staining of the tumorigenesis experiment also confirmed that DMKG and radiotherapy promoted the expression of PD-L1 in the tumor (Additional file 2: Figure S2).

### DMKG combined with radiotherapy inhibited tumor growth and promoted the therapeutic effect of PD-L1 inhibitors

**Fig. 6:**
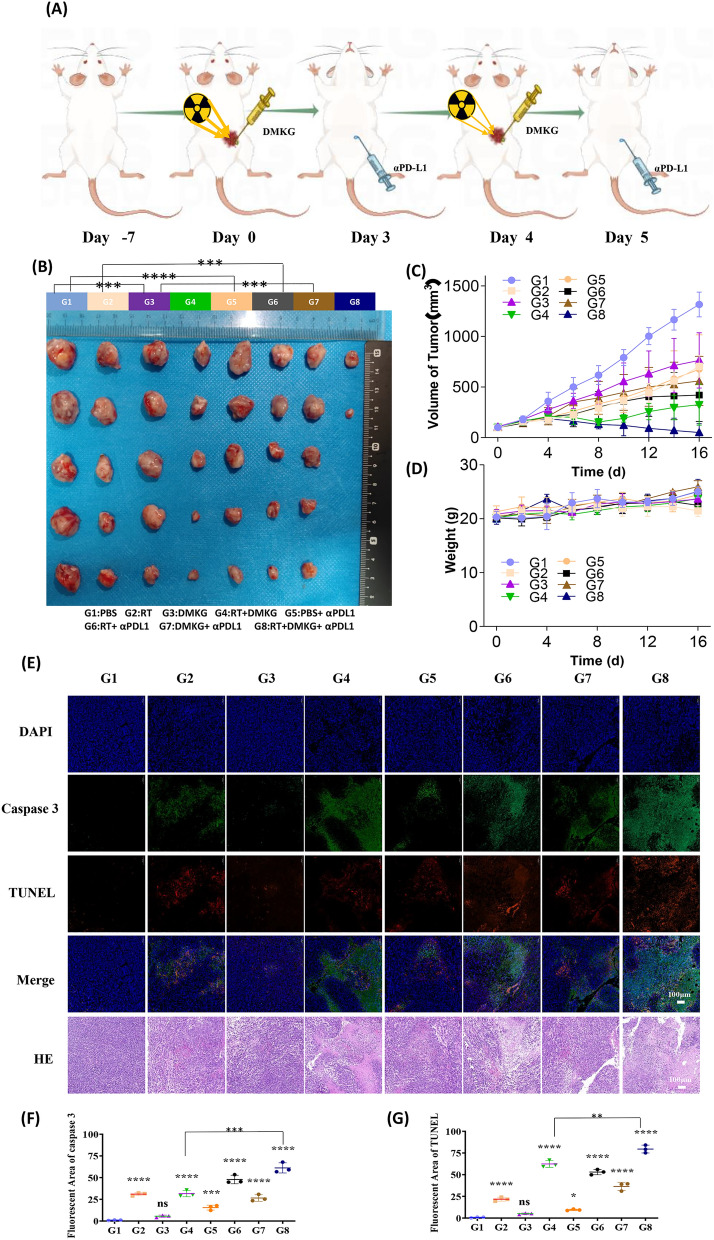
**A** Overall experimental timeline of the in vivo antitumor experiment (n = 5). **B** Pictures of tumors harvested from mice. **C** The 4T1 tumor growth curves after different treatments. **D** The body weight of mice during the treatment. **E** H&E, TUNEL, and Caspase 3 staining of harvested tumors after different treatments. **F** Quantitative analysis of Caspase 3 staining. **G** Quantitative analysis of TUNEL staining

To evaluate whether the results in vitro could be reproduced in vivo, we established subcutaneous tumors using 4T1 cells and further investigated the role of DMKG plus radiotherapy combined with PD-L1 inhibitors in tumor therapy. The treatment is shown in Fig. 6A. Tumorigenesis experiments showed that DKMG combined with radiotherapy had a good inhibitory effect on tumors, and coupled with a PD-L1 inhibitor, significantly inhibited tumor growth (Fig. 6B, C). The body weight of the mice did not change significantly during the treatment (Fig. 6D). The immunofluorescence staining analysis of the tumor tissue showed that DMKG combined with radiotherapy can promote tumor area apoptosis and that the expression of caspase3, and combined with anti PD-L1 results in more marked tumor apoptosis (Fig. 6E–G).

### DMKG combined with radiotherapy affected the infiltration of immune cells in the tumor area

**Fig. 7:**
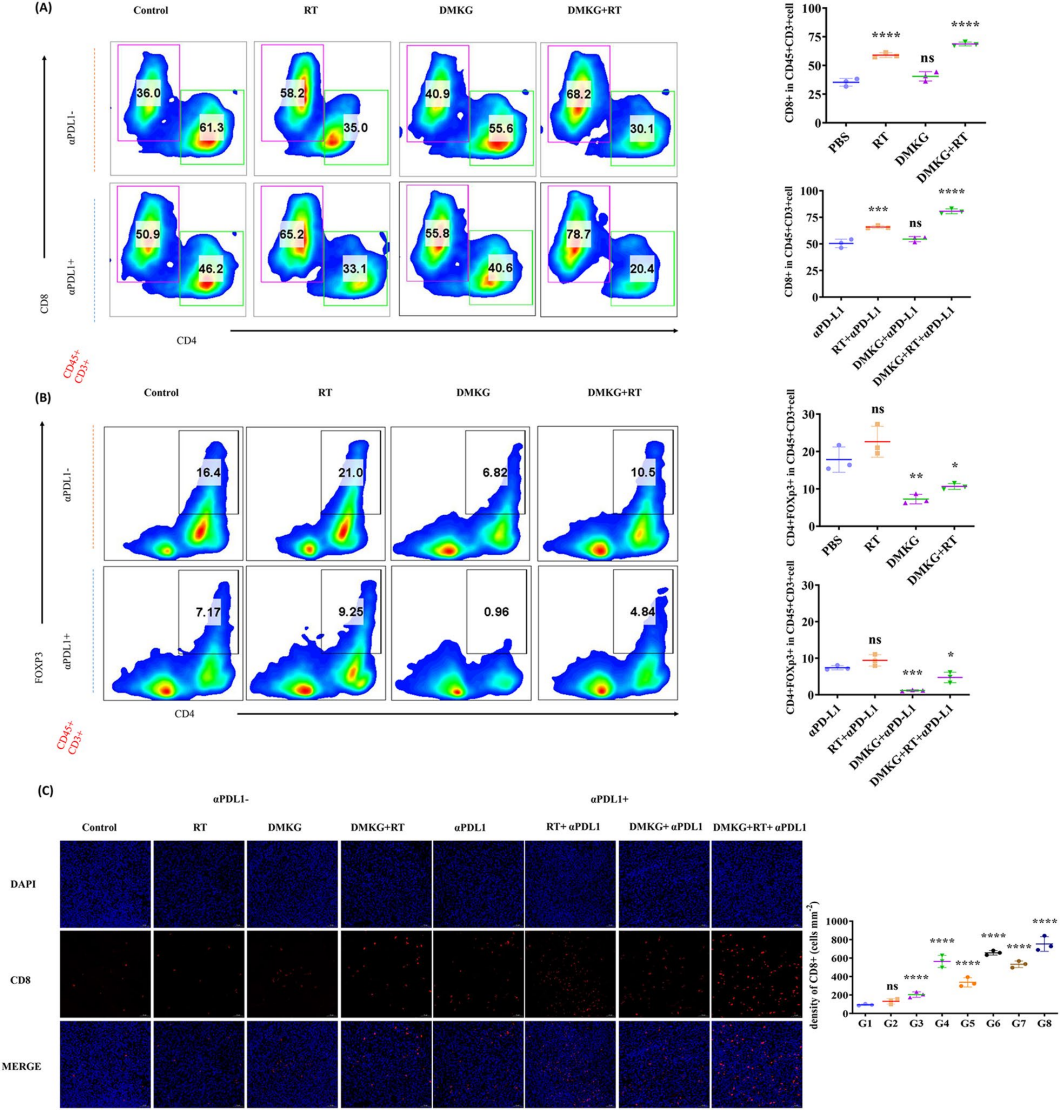
**A** Flow cytometry analysis of CD4 + and CD8 + T cells gating on CD45 + CD3 + cells. CD8 + T cells were quantified under different treatments. **B** Flow cytometry analysis of CD4 + and FOXP3 + T cells gating on CD45 + CD3 + cells, and the levels of Treg cells under different treatments were quantitatively analysed. **C** Immunofluorescence staining of CD8 in tumors from different groups

Tumorigenesis experiments have shown the inhibitory effects of PD-L1 inhibitors combined with radiotherapy and DMKG on tumor, and we wanted to further study whether these effects are related to the activation of immunity. In order to explore the changes in the tumor immune microenvironment, we used flow cytometry to detect immune cell infiltration and activation. Results showed that DMKG and radiotherapy increased CD8 + T cell infiltration significantly (Fig. 7A). Although radiotherapy can increase the number of Treg cells in the tumor area, DMKG significantly inhibits the number of Treg cells infiltrating the tumor. At the same time, PD-L1 inhibitors significantly reduced the tumor infiltration of Treg cells (Fig. 7B). The changes in the immune microenvironment were further verified by immunofluorescence staining of CD8 + cells in tumor (Fig. 7C).

### DMKG combined with radiotherapy promoted the activation of CD8 + T cells in the tumor area and had a synergistic effect with immunotherapy

**Fig. 8:**
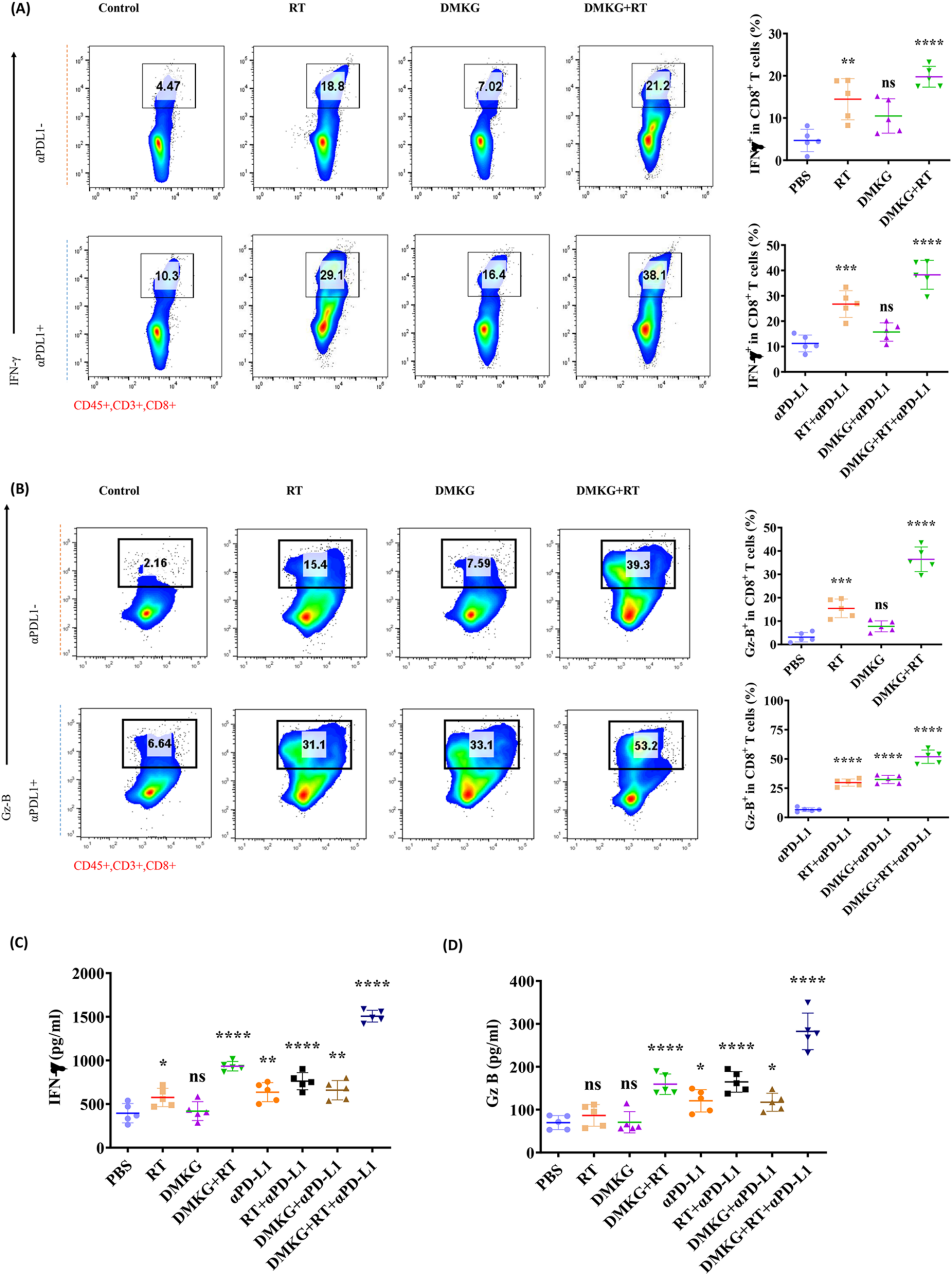
**A** Flow cytometry analysis of IFN-γ + cells gating on CD45 + CD3 + CD8 + T cells; IFN-γ + of CD8 + T cells were quantified under different treatments. **B** Flow cytometry analysis of Gz-B + cells gating on CD45 + CD3 + CD8 + T cells; Gz-B + of CD8 + T cells were quantified under different treatments. ELISA showed the levels of IFN-γ (**C**) and Gzms-B (**D**) in tumor tissues under different treatments

To observe the effect of DMKG and radiotherapy on immune cell function, the activation of CD8 + T cells was also examined. Flow cytometry results showed that DMKG and radiotherapy activated CD8 + T cells. After combination with anti-PD-L1, T cells were significantly activated, especially GZMB + CD8 + T cells (Fig. 8A, B). Finally, we detected the levels of IFN-γ and GzB in tumor tissue, and the results also showed that combined treatment with PD-L1 significantly increased the levels of IFN-γ and GzB in tumor (Fig. 8C, D).

## Discussion

Triple-negative breast cancer (TNBC) represents an extremely malignant subtype of breast cancer. Owing to the absence of corresponding targeted therapies and the notable resilience of TNBC to radiotherapy and chemotherapy, the survival outcomes for TNBC patients remain distressingly limited. Despite the promising potential of immunotherapy, the patient response rate continues to be suboptimal, and the clinical efficacy of combined radiotherapy and immunotherapy is yet to achieve satisfactory outcomes.This shortcoming might be attributable to the radioresistance of TNBC, which hinders the infiltration of immune cells into the tumor, thereby precluding the establishment of a local proinflammatory microenvironment and leading to diminished therapeutic efficacy. Thus, enhancing radiosensitivity and reprogramming the tumor microenvironment emerge as pivotal strategies to surmount the treatment barriers associated with TNBC.

Radiotherapy is a prevalent modality for the treatment of various solid malignancies, with its role in tumor suppression and immune modulation being a focal point of extensive research. Numerous investigations have been directed towards the identification of radiotherapy sensitizers to amplify therapeutic efficacy; however, a significant number of clinically-employed agents exhibit high toxicity and severe adverse effects [28]. In this study, we employed dimethyl alpha-ketoglutarate (DMKG), a precursor of AKG, to enhance the impact of radiotherapy-induced nuclear damage and intracellular reactive oxygen species accumulation. It has been demonstrated that radiation induces immunogenic cell death (ICD) in tumor cells, consequently releasing an abundance of tumor-associated antigens which can stimulate T cells via the activation of antigen-presenting cells [29, 30]. Nonetheless, radiotherapy also results in the upregulation of immune checkpoints and the recruitment of immunosuppressive cells [31], thereby underscoring the significance of combining radiotherapy with immune regulation. In this study, we found that DMKG can significantly promote ICD caused by radiotherapy, and Treg cells in the tumor area are significantly reduced when DMKG is used. Additionally, existing literature suggests that AKG may modulate the differentiation of Treg cells towards a proinflammatory direction [32]. The hepatic and renal function of mice remained unaffected during in vivo treatment, as depicted in Additional file 3: Figure S3, which implies that the combination of DMKG with radiotherapy might effectively remodel the tumor immune microenvironment.

Autophagy, a mechanism of cellular death, holds a vital position in the management of malignancies. On one hand, autophagy can potentially bolster the efficacy of immunotherapy by facilitating PD-L1 degradation; conversely, it can lead to immune evasion through the degradation of MHC-I/II [15, 33]. Hence, autophagy appears to perform a bidirectional role in the regulation of tumor immunity.Numerous studies corroborate the notion of autophagy functioning as a double-edged sword in oncology, wherein it suppresses tumors during the early stages of their development but mediates drug resistance in later stages [34, 35]. Studies have found that the autophagy of tumor cells increases after radiotherapy, and autophagy may also be a mechanism of tumor radiotherapy tolerance [14]. Additionally, evidence suggests that autophagy inhibition can enhance immunotherapy [36], indicating that modulating radiation-induced autophagy might potentiate tumor radiosensitivity [37, 38]. Our findings demonstrate that DMKG can impede the formation of radiation-induced autophagosomes and inhibit cellular autophagy. By promoting immunogenic cell death and curtailing autophagy, increased tumor cell death may result in the release of a higher volume of proinflammatory factors, crucial for the recruitment and activation of antitumor T cells. Prior research has identified CCL5 and CXCL10 as T cell activators, in addition to activating CD4 + T cells, NK cells, and M1 macrophages in tumors [16, 39], Consequently, we assessed the levels of CCL5 and CXCL10 under different treatment conditions. Our findings indicate that the combination of DMKG with radiotherapy markedly boosts the release of CCL5 and CXCL10. The proinflammatory effect of combining DMKG with radiotherapy was substantiated by in vitro dendritic cell maturation assays and in vivo immunoassays.

A myriad of factors influences the efficacy of immunotherapy. Beyond immune cell infiltration, the expression of PD-L1 on tumor cells has been associated with the effectiveness of immunotherapy [40, 41]. Our results show that DMKG and RT can activate STAT1, thereby promoting PD-L1 expression. Concurrent studies have shown that the activation of STAT1 promotes PD-L1 expression [26], potentially elucidating the mechanism by which DMKG and RT influence PD-L1 expression. Moreover, the activation of STAT1 has been reported to facilitate the transmission of tumor antigens and the activation of tumor immunity [42], which may also be the possible reason for the improvement of the immune microenvironment by DMKG and RT. In addition to constitutive expression, interferon gamma (IFN-γ) can induce PD-L1. T cells infiltrating the tumor can secrete IFN-γ, resulting in PD-L1 upregulation [43, 44]. Oncogenic MYC and RAS regulate PD-L1 transcription and RNA stability [45, 46], CMTM4 and CMTM6 proteins also affect the stability of PD-L1 [47, 48]. It has been demonstrated that immunotherapy response often correlates with induced PD-L1 expression, and the IFN-γ pathway has been associated with patient resistance to PD-1 therapy [49]. Therefore, maintaining the expression of PD-L1 induced by exogenous IFN-r stimulation may be integral to successful immunotherapy. Our results demonstrate that DMKG and radiotherapy significantly enhance the infiltration of IFNγ + CD8 + T cells into the tumor area and augment PD-L1 expression in the tumor vicinity. While high PD-L1 expression suppresses tumor immunity, it can be modulated using PD-L1 inhibitors, possibly promoting the efficacy of PD-L1 inhibitors. CD8 + T cells can secrete IFN-γ to destroy tumors, and another crucial mechanism involves perforating tumor cells and mediating tumor cell death through granzyme B, which primarily functions through caspase-3 [50]. As an activation marker for CD8 + T-cells, granzyme B is also significant. Our results confirmed that GZMB + CD8 + T cells markedly increased in tumor tissues following the addition of a PD-L1 inhibitor, and immunostaining of tumor sections also displayed enhanced caspase-3 expression. Given these findings, it is suggested that granzyme B might contribute to the impact of immunotherapy in this study when DMKG and radiotherapy are used in combination (Additional file 5).

In summary, our findings propose that the supplementation of alpha-ketoglutarate (AKG) augments radiotherapy sensitization and ameliorates the tumor immune microenvironment. This could be associated with the inhibition of autophagy and the stimulation of inflammatory cytokine release. Owing to its robust biosafety profile, DMKG presents promising prospects for clinical translation, potentially enhancing radiotherapy sensitivity and improving the outcomes of immunotherapy.

## Supplementary Information

**Additional file 1: Figure S1.** Clonal formation of breast cancer cells under different treatments.

**Additional file 2: Figure S2.** Expression of PD-L1 in tumor regions under different treatments.

**Additional file 3: Figure S3.** Serum levels of AST, ALT, BUN and CRE in mice during treatment. AST: aspartate aminotransferase. ALT: Alanine transaminase. BUN: Blood Urea Nitrogen. Cre: serum creatinine.

**Additional file 4: FigureS4.** Infiltration and activation of DC cells in the tumor area under different treatments.

**Additional file 5:** PCR primer and reagents used in this study.

## Data Availability

All data and materials supporting the conclusions are included in the main paper, and the corresponding authors can be contacted for more detailed methods and raw results.

## Abbreviations

RT: Radiotherapy
DC cell: Dendritic cell
PD-L1: Programmed cell death-ligand 1
TNBC: Triple-negative breast cancer
α-KG: Alpha-ketoglutarate
ROS: Reactive oxygen species
HMGB1: High mobility group protein 1
CRT: Calregulin
IDO: Indoleamine-2,3-dioxygenase
