# Supplementary figures and images for: Ketoglutaric acid can reprogram the immunophenotype of triple-negative breast cancer after radiotherapy and improve the therapeutic effect of anti-PD-L1
Source: J Transl Med. 2023 Jul 12;21:462. doi: 10.1186/s12967-023-04312-2 (PMC10337087; doi:10.1186/s12967-023-04312-2)

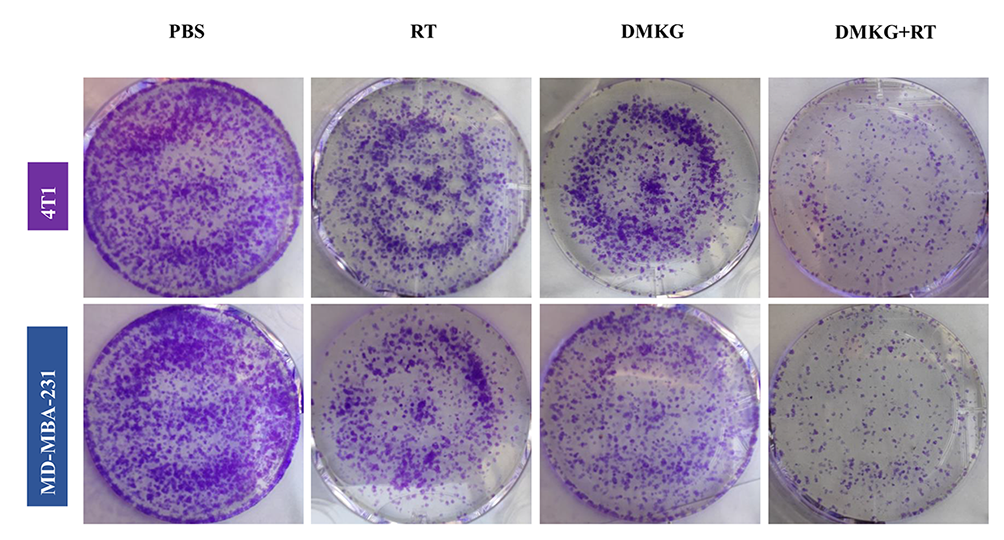

Supplement: Supplementary file 1 — Additional file 1: Figure S1. Clonal formation of breast cancer cells under different treatments. [file 12967_2023_4312_MOESM1_ESM.tif]

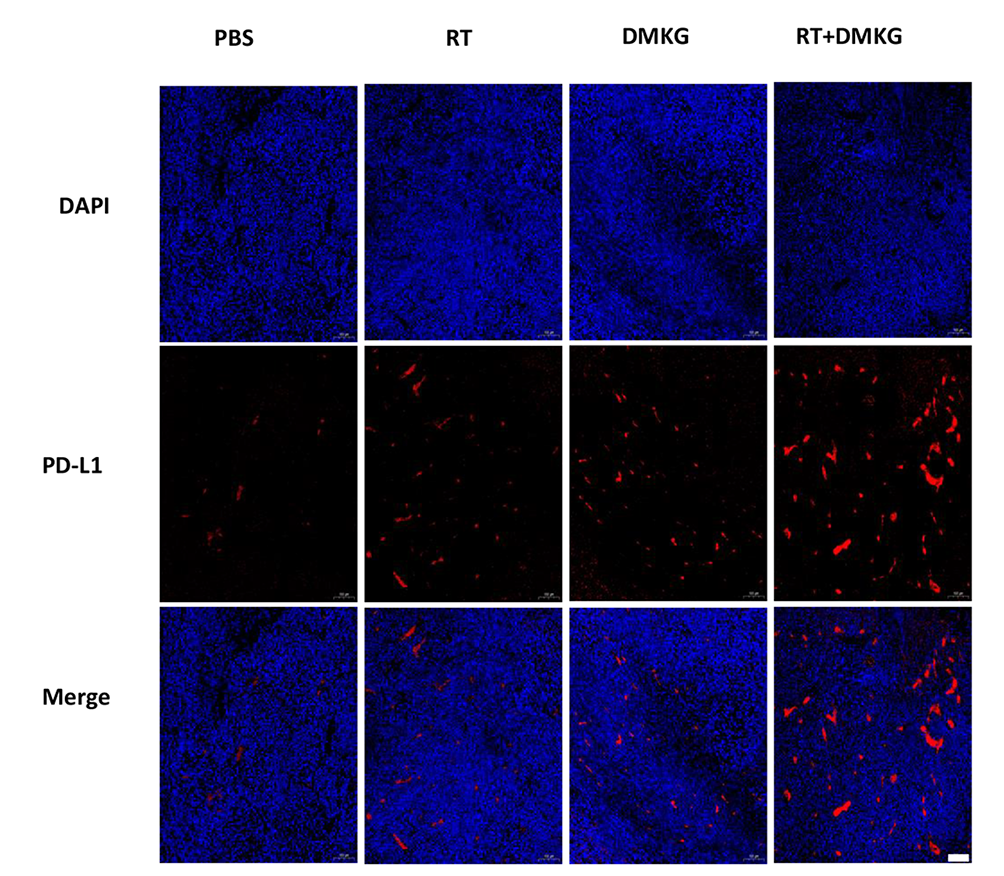

Supplement: Supplementary file 2 — Additional file 2: Figure S2. Expression of PD-L1 in tumor regions under different treatments. [file 12967_2023_4312_MOESM2_ESM.tif]

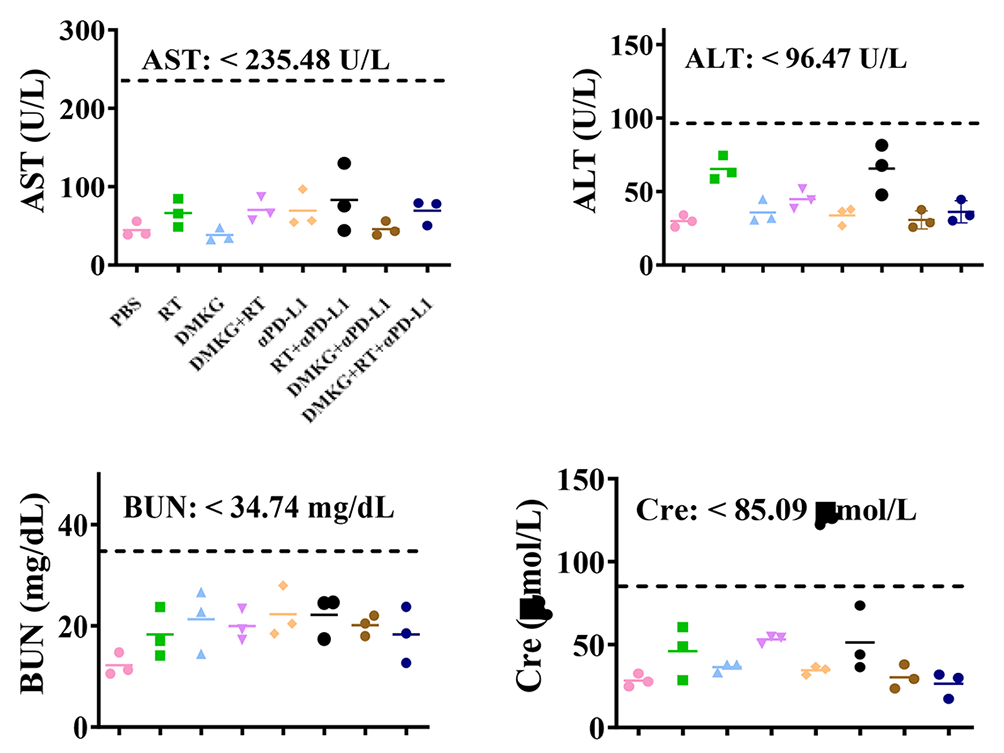

Supplement: Supplementary file 3 — Additional file 3: Figure S3. Serum levels of AST, ALT, BUN and CRE in mice during treatment. AST: aspartate aminotransferase. ALT: Alanine transaminase. BUN: Blood Urea Nitrogen. Cre: serum creatinine. [file 12967_2023_4312_MOESM3_ESM.tif]

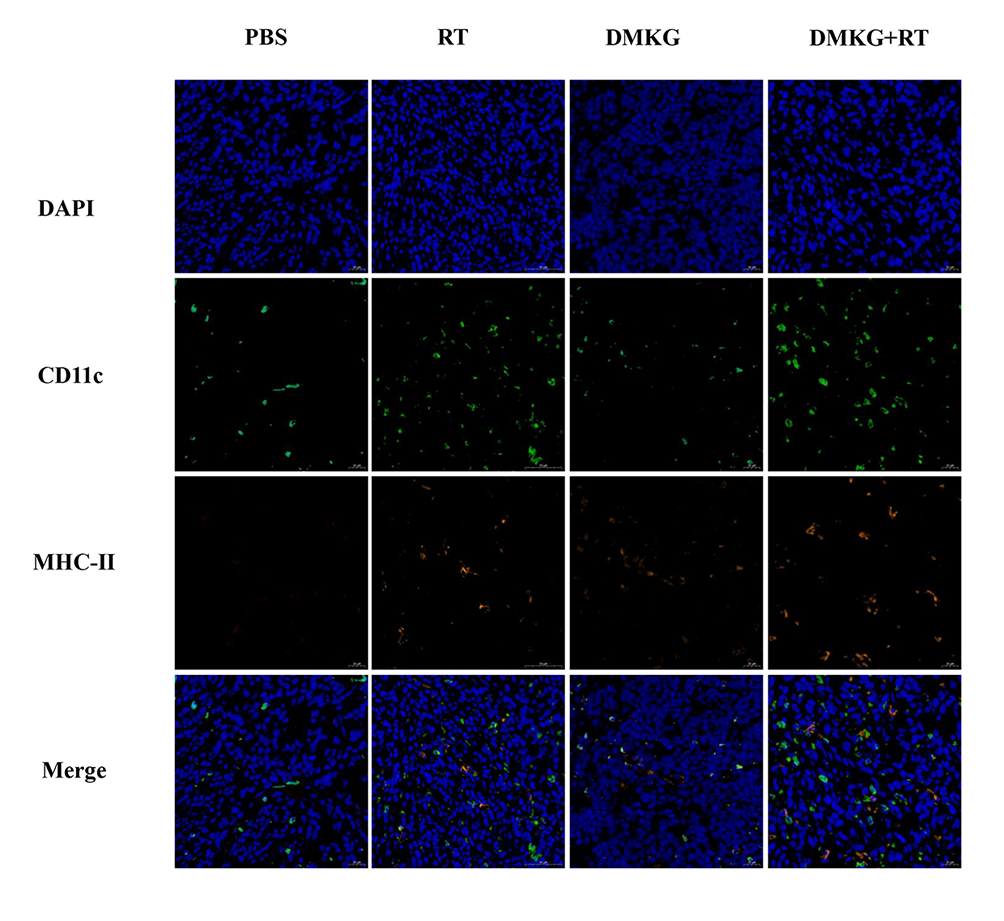

Supplement: Supplementary file 4 — Additional file 4: FigureS4. Infiltration and activation of DC cells in the tumor area under different treatments. [file 12967_2023_4312_MOESM4_ESM.tif]
