# Supplementary material for: Ketoglutaric acid can reprogram the immunophenotype of triple-negative breast cancer after radiotherapy and improve the therapeutic effect of anti-PD-L1
Source: J Transl Med. 2023 Jul 12;21:462. doi: 10.1186/s12967-023-04312-2 (PMC10337087; doi:10.1186/s12967-023-04312-2)
Supplement: Supplementary file 5 — Additional file 5: PCR primer and reagents used in this study. [file 12967_2023_4312_MOESM5_ESM.docx]

**PCR primer sequence**

H-CCL5

F CCTCGCTGTCATCCTCATTGCT

R ATCCTTGACCTGTGGACGACT

M-CCL5

F CGCCAAGTGTGTGCCAACCC

R CCCATTTTCCCAGGACCGAGT

H-CXCL10

F ACCCTGGCACTATAATGTAAGCTC

R TTTGAAGCAGGGTCAGAACATCCAC

M-CXCL10

F ATCATCCCTGCGAGCCTA

R AGGCTCTCTGCTGTCCATC

H-PD-L1

F TTGCTGAACGCCCCATACAA

R GTCCAGATGACTTCGGCCTT

M-PDL1（CD274）

F AAAGACGAGCATAGCCGAAC

R GCCACACCAATCCAACACC

H-IDO

F CATCCTGATTCCTGCAAGCC

R CTCTGCTATGATAAAATGTGCTCT

M-IDO

F CCTTGCTACAAGTGTCCTGGC

R AGGTGGTCAGTTCCACATTCG

H-Ctla4

F CCCCAACAGAGCCAGAATGT

R TGGGTTCCGCATCCAACTTT

M-CTLA-4

F AAAGTCCCCGAGTCTGTGTG

R CGGAGTCCAAGACAAGCCAT

**Information about the antibodies used in the experiments**

| index | Item No | COMPANY |
| --- | --- | --- |
| caspase1 | 22915-1-AP | proteintech |
| caspase3 | 19677-1-AP | proteintech |
| caspase8 | ab25901 | abcam |
| HMGB1 | AF300121 | AiFang biological |
| calregulin | AF04178 | AiFang biological |
| Beclin1 | 11306-1-AP | proteintech |
| LC3 I/II | ab128025 | abcam |
| 4E-BP | 9644 | CST |
| P-4E-BP | 2855 | CST |
| S6K | 14485-1-AP | proteintech |
| P-S6K | 28735-1-AP | proteintech |
| MTOR | 66888-1-Ig | proteintech |
| P-MTOR | 67778-1-Ig | proteintech |
| Stat1 | ab109320 | abcam |
| P-stat1 | ab109461 | abcam |
| PD-L1 | 66248-1-Ig | proteintech |
| β-actin | 66009-1-Ig | proteintech |
| CD11c-BV421 | 565452 | BD Pharmingen |
| CD40-PE | 5537911 | BD Pharmingen |
| CD80-APC | 104713 | Biolegend |
| CD86-BV650 | 564200 | BD Pharmingen |
| MHCII-PerCP-CY5.5 | 746070 | BD Pharmingen |
| CD45-Alexa700 | 560510 | BD Pharmingen |
| CD3-BV510 | 563024 | BD Pharmingen |
| CD4-BV605 | 563151 | BD Pharmingen |
| CD8a-APC-Cy7 | 557654 | BD Pharmingen |
| CD25-BB515 | 564424 | BD Horizon |
| FOXP3-BV421 | 562996 | BD Horizon |
| CD274-PE | 558091 | BD Pharmingen |
| CD274-FITC | 558065 | BD Pharmingen |
| IFN-γ-APC | 17–7311-82 | Thermo |
| GzB- Alexa 645 | 50590S | Thermo |

**Information about the ELISA kit used in the experiments**

Mouse CCL5 ELISA Kit（KE10017）,

Human CCL5 ELISA Kit(KE00093),

(IP-10/CXCL10)ELISA Kit(CSB-E08181h)( CSB-E08183m),

IFN-γ ELISA Kit(CSB-E04578m),

GZMB ELISA Kit(CSB-E08720m),

HMGB-1 elisa kit(PH406),

ATP Assay Kit (Colorimetric/Fluorometric)( ab83355).

**Tumor dissociation kit,mouse: Miltenyi, order No. 130-096-730**
